# Deep Penetrating Nevus and Borderline-Deep Penetrating Nevus: A Literature Review

**DOI:** 10.3389/fonc.2020.00837

**Published:** 2020-05-20

**Authors:** Ioana Cosgarea, Klaus G. Griewank, Loredana Ungureanu, Arturo Tamayo, Timo Siepmann

**Affiliations:** ^1^Department of Dermatology, Translational and Clinical Research Institute, Newcastle University, Newcastle upon Tyne, United Kingdom; ^2^Division of Health Care Sciences, Center for Clinical Research and Management Education, Dresden International University, Dresden, Germany; ^3^Department of Dermatology, University Hospital Essen, West German Cancer Center, University Duisburg-Essen and the German Cancer Consortium (DKTK), Essen, Germany; ^4^Dermatopathologie bei Mainz, Nieder-Olm, Germany; ^5^Department of Dermatology, Iuliu Haţieganu University of Medicine and Pharmacy, Cluj-Napoca, Romania; ^6^The Max Rady Faculty of Health Sciences, University of Manitoba and Winnipeg, Brandon Regional Hospital, Winnipeg, MB, Canada; ^7^Department of Neurology, University Hospital Carl Gustav Carus, Technische Universität, Dresden, Germany

**Keywords:** deep penetrating nevus, DPN, borderline/atypical deep penetrating nevus, DPN treatment, borderline/atypical deep penetrating nevus treatment

## Abstract

Deep penetrating nevi (DPN) are rare melanocytic nevi, which can exhibit atypical histological features hampering the differentiation from malignant melanoma. DPN are considered benign melanocytic lesions, but rare spread to lymph nodes and unfavorable clinical outcomes associated with borderline/atypical DPN (B-DPN) has been reported. Since no guidelines are available for DPN and B-DPN, we aimed to review the literature on DPN and B-DPN to assess the management and prognosis. We screened 3,513 references from EMBASE, Scopus and Medline databases, and included 15 studies with a total of 355 DPN patients and 48 B-DPN patients. Therapeutic interventions ranged from simple excision to wide excisions and sentinel lymph node biopsy (SLNB), with block lymph node dissection in some positive SLNB cases. Follow-up periods ranged from 3 months to 23 years during which a total of five recurrences, two in DPN and three in B-DPN group, and three metastases, in B-DPN group, were reported. While some of the included studies comprised clinical and histopathological correlation, few included genetic assessment. The present review highlights the need for prospective cohort studies applying composite measures to identify effective regimens of diagnostic workup and treatment in DPN and B-DPN.

## Introduction

**Figure 1:**
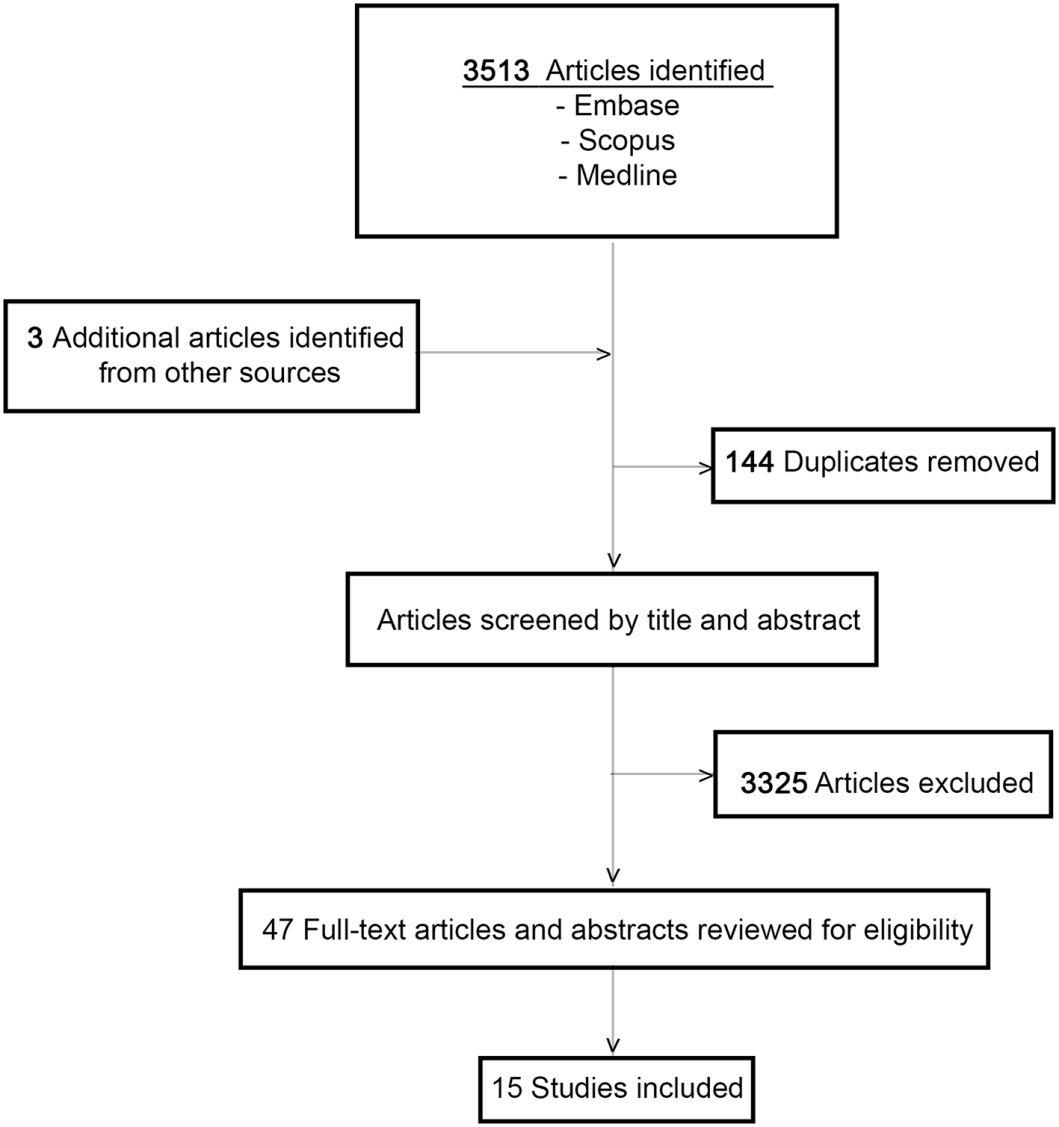
Study selection flowchart.

The term deep penetrating nevus (DPN) was first coined by Seab et al., in 1989, the author describing a series of cases which were initially misdiagnosed as malignant melanoma (1). Due to its clinical and, in particular, its histopathological appearance, DPN can be challenging to differentiate from cellular blue nevi, Spitz nevi, and malignant melanoma (2). Clinically presenting as solitary, pigmented papules, or nodules, DPN is more common in young individuals with a prevalent appearance in the head and neck area (3, 4). Histologically it is characterized as a well-demarcated, wedge-shaped lesions reaching down into the reticular dermis/subcutis exhibiting epithelioid/spindle-cell melanocytic nests with low-grade cytologic atypia and possible mitotic figures (1, 2, 5, 6). Despite the conspicuous histopathologic appearance, DPNs have a good prognosis demonstrating a benign behavior (1, 2, 5, 6). However, spread to regional lymph nodes has been described (7) and even cases of metastasis from DPN with atypical features classified as “borderline” (B-DPN) can be found in the literature (7–10). Representing a diagnostic challenge, even to experts of the dermatopathology and pathology field (7), the correct diagnosis is paramount to effective clinical management and outcome. Recommendations for therapeutic management and follow-up are not consistent and not based on sufficient clinical data. Under-treatment and over-treatment including prolonged follow-up, is common in patients with these tumors (2, 6, 8, 9, 11, 12). We aimed to provide a review of the literature on DPN and B-DPN assessing clinical management including treatment and follow-up evaluation.

## Materials and Methods

We conducted a review of the Medline, Scopus, and Embase datasets for clinical studies published from 1989 to July 30, 2019, on DPN and B-DPN.

The words “deep penetrating nevus” and “deep nevus” were used to identify studies examining patients with this condition containing clinical and laboratory workup data they underwent. Combinations of MeSH (Medical Subject Heading) terms and boolean operators applied in our search on Medline included are listed in the following: deep penetrating nevus OR deep penetrating nevus OR deep blue nevus OR deep blue nevus OR deep nevus OR deep nevus. Scopus was searched using the following combinations of MeSH terms and boolean operators: [(nevus) OR (Nevi) OR (Nevus) OR (Nevi)] AND [(deep) OR (blue) OR (penetrating)]. Combinations of MeSH terms and boolean operators used in our search on Embase included the following: [nevus/nevus AND ((blue) OR (deep)]. We only considered articles published in the English or German language for further review and excluded duplicates. In order to address the anticipated overall lack of well-designed studies in large populations of patients with DPN and B-DPN we included prospective studies of any design, retrospective cohort analyses as well as case reports. However, in order to be able to evaluate sustained effects of clinical management and prognostic outcomes we only included those studies that contained follow-up data. Articles were screened based on title and abstract to determine eligibility for our review. Entire articles were reviewed and retrieved to assess acceptability. Furthermore, we manually searched the bibliographies of included articles.

## Results

The initial search identified a total of 3,513 studies and during the review process a further three articles were identified by manual searching of the reference lists. After duplicate articles were eliminated, a total of 3,369 articles remained. Papers were narrowed by title, abstract, and full-text review. A total of 15 articles, met the inclusion criteria (Figure 1). All 15 papers that met the inclusion criteria are listed in Tables 1A,B. The studies were categorized into articles reporting on clinical management of DPN and those reporting on B-DPN. The included studies consist of 10 cohort studies and five case reports including a total of 355 patients with DPN and 48 patients with B-DPN. The clinical characteristics and follow-up data of the studies included are shown in Tables 1A,B.

**Table 1A:** Clinical data of patients included in the DPN group.

**ID**	**No. of cases**	**Gender (No.)**		**Age, range (mean), years**	**Location (No.)**						**Follow-up, range**	**Local recurrence (No.)**	**Metastasis (No.)**	**Source**
		**Male**	**Female**		**Head and neck**	**Trunk**	**Upper extremity**	**Lower extremity**	**Other**	**Unknown**				
1	70	33	37	3–63 (NA)	29	8	15	12	6	0	1–23 y	0	0	(1)
2	10	5	5	u−49 (22)	2	4	4	0	0	0	4–13 y	0	0	(11)
3	41	15	26	4–47 (25)	13	11	11	6	0	0	4–76 m	0	0	(6)
4	14	7	7	17–36 (26)	6	1	6		0	1	1/2–8 y	0	0	(13)
5	31	14	17	3–56 (26)	12	7	2	7	0	3	1–17 y	1	0	(2)
6	146	61	85	4–64 (28)	52	51	28	15	0	0	1/2–13 y	1	0	(12)
7	1	1	0	3 m	0	0	0	1	0	0	15 m	0	0	(14)
8	1	1	0	59	0	0	1	0	0	0	202 m	0	0	(15)
9	1	0	1	56	0	0	0	1	0	0	4 y	0	0	(16)
10	1	0	1	49	0	0	0	0	1 (buccal mucosa and soft palate)	0	1 y	0	0	(17)
11	5	0	5	26–56 (41)	3	0	0	2	0	0	30 m	0	0	(18)
12	34	15	19	7–51 (25)	0	0	0	0	34 (conjunctiva)	0	0.3–16.3 y	0	0	(19)
**Total**	355 (100%)	152 (42.8%)	203 (57.1%)	3 m−64 y	117 (32.9%)	82 (23%)	111 (31.2%)	111 (31.2%)	41 (11.5%)	4 (1.1%)	4 m−23 y	2	0	

*Twelve studies were included in the DPN group. Information on clinical characteristics as well as follow-up data was extracted from each study and is presented in the table. NA, indicates not available; m, months; y, years*.

**Table 1B:** Clinical data of patients included in the B-DPN group.

**ID**	**No. of cases**	**Gender (No.)**		**Age, range (mean), years**	**Location (No.)**						**Lymph node involvement and further treatment**	**Follow-up, range**	**Local recurrence (No.)**	**Metastasis (No.)**	**Source**
		**Male**	**Female**		**Head and neck**	**Trunk**	**Upper extremity**	**Lower extremity**	**Other**	**Unknown**					
1	7	5	2	14–36 (22.3)	1	2	4	0	0	0	Four patients had positive sentinel lymph nodes (SLN). Patients underwent completion lymphadenectomy with no further evidence of disease and received adjuvant therapy with interferon	4 y	1	1	(9)
2	40	16	24	10–62 (34.5)	14	12	10	4	0	0	Nineteen patients underwent sentinel lymph node biopsy with positive SLN in seven cases. 4/7 underwent completion lymphadenectomy with no further evidence of disease. Two patients received adjuvant interferon alpha therapy	5 m−5.42 y	3	2	(8)
3	1	1	0	4	1	0	0	0	0	0	Initially, no lymphadenopathy. After 4 months—excision of a visible and palpable lymph node with pigmented atypical cells followed by a type III modified radical neck dissection with removal of 39 lymph nodes which were negative	8 m	0	0	(20)
**Total**	48 (100%)	22 (45.8%)	26 (54.1%)	4–62 y	16 (33.3%)	14 (29.1%)	14 (29.1%)	4 (8.3%)	0	0	–	5 m−5.42 y	4	3	

*Three studies were included in the B-DPN group. Information on clinical characteristics and treatment management, as well as follow-up data was extracted from each study and is presented in the table. M, months; y, years*.

### Clinical Characteristics

For the deep penetrating nevus group the age range in studies, excluding case report, varied from 3 months to 64 years with a mean age of 30 years, similar to the B-DPN group where the age range was 4–62 years with the mean age in all studies being under 30 years. The gender distribution was similar for both groups with a slightly higher number of females, 203 (57.1%) in DPN and 25 (54.1%) in B-DPN, than males, 152 (42.8%) in DPN, and 22 (45.8%) in B-DPN. The location of the nevus was divided into six categories: head and neck, trunk, upper extremity, lower extremity (including buttocks), other, and unknown. Most nevi in the DPN group were located in the head and neck area, 117 (32.9%), and on the upper and lower extremity, 111 (31.2%). Out of the 111 nevi on the extremities, more nevi were diagnosed on the upper extremity than on the lower one, 61 vs. 44, respectively. Among unusual nevi locations was the conjunctiva, with 34 DPN cases (19), and the buccal mucosa and soft palate in one case (17). In the B-DPN group the most frequent location was similar to the DPN group, head and neck with 16 cases followed by the trunk and upper extremity, each with 14 cases, and in the remaining four cases the location being the lower extremity.

### Management

Information regarding treatment management for the DPN group was not available for all the included studies. Simple excision without any further surgical procedures was the main treatment the majority of the patients received. Several cases underwent re-excision, in cases were the nevus was not completely removed the first time, and some patients underwent wide-excision, without further specifications in this regard. For the B-DPN group, the treatment varied from excisions/wide excisions to sentinel lymph node biopsies (SLNB) and systemic treatment. The 2010 study by Magro et al., included 32 patients with borderline melanocytic tumors out of which seven patients had the diagnosis of a B-DPN (9). Four of those patients had positive sentinel lymph node biopsies (SLNB). One of those patients was initially diagnosed as DPN and the lesion recurred after 1.5 years showing histologic characteristics of a deeply invasive melanoma. A re-evaluation of the initial biopsy diagnosed the lesion as a B-DPN, which in the eyes of the authors would have had the indication for wide excision and SLNB. The patient developed subsequent multi-organ metastasis and died. The other patients with positive SLB underwent completion lymphadenectomy with no further evidence of disease and received adjuvant therapy with interferon. Patients did not show any further recurrences or metastasis within a 4-year follow-up. The second study by Magro et al. (8) included 40 patients with a DPN-like borderline tumor. In 35 cases a re-excision was performed and in 23 of these cases the procedure was a wide re-excision, residual borderline tumor being present in 11 samples. From the 23 patients with wide re-excision 19 underwent SLNB out of which 7 were positive. The authors reported that in most of these positive SLNB cases, the tumor deposits were subcapsular and small, while in one patient, who presented a 1-year recurrence post-treatment there were extensive parenchymal deposits. Complete lymphadenectomy was performed in four of the patients with positive SLNB with no further evidence of disease and two patients received adjuvant therapy with interferon alpha. In two of the 40 cases, the initial diagnosis was of a dysplastic nevus and a cellular blue nevus, the patients receiving small conservative re-excision or no re-excision, respectively. Both patients developed plexiform melanomas in the vicinity of the previous lesion with metastatic disease due to which both patients died. One case report was included in this group, a 4-year-old boy with a lesion on his neck diagnosed as DPN who developed a visible and palpable lymph node 4 months after treatment. SLNB revealed pigmented atypical cells and the boy underwent comprehensive type II modified radical neck dissection with the removal of 39 lymph nodes, all negative. Staging revealed no further evidence of metastasis. Re-evaluation of the primary biopsy led to re-diagnosis of the lesion as a malignant melanoma.

### Follow-Up

The follow-up of the patients in the DPN group ranged from 4 months to 23 years. In five studies, some patients had a follow-up that lasted longer than 10 years. Out of the 12 studies, local recurrences were reported just in two patients from two different studies and no cases of metastasis were observed. Follow-up in the B-DPN group ranged from 5 months to 5 years. A total of four recurrences and three deaths were observed, all three cases being misdiagnosed initially which lead to an undertreatment of the patients.

## Discussion

The histologic diagnosis of atypical melanocytic tumors can be very challenging even for experienced dermatopathologists and pathologistis (7) and differentiating between a benign or malignant lesions has therapeutic and prognostic consequences. Despite the fact that DPN has been vastly described as a benign melanocytic lesion (1, 6, 11, 13, 18, 19) its malignant potential has being discussed intensely (5, 7) and cases with regional lymph node involvement have been reported (2, 5, 12). However, the 2009 International Melanoma Pathology Study Group agreed that distant disease beyond the regional nodes is rare and that in the absence of mitotic activity, nuclear pleomorphism, and expansive growth, <2% of the lesions spread to the lymph node (5). During the 2008 “XXIX Symposium of the International Society of Dermatopathology in Graz” expert dermatopathologistis and pathologists reviewed 57 cases of MELTUMP (Melanocytic Tumors of Uncertain Malignant Potential) which included atypical Spitz tumors (AST) and atypical epithelioid/spindled blue nevi, the latter category including DPNs (7). The patients were stratified in three groups with a favorable (no metastatic disease during a 5 year follow-up), unfavorable (tumor-related death and/or lymph node and/or visceral metastasis), and borderline (nodal tumor cell deposits ≤ 0.2 mm) behavior and the authors noted that the unfavorable group presented more frequently three histopathologic features, namely presence of mitosis, mitosis near the base, and inflammatory reaction. Furthermore, the authors commented on the difficulty of reaching a consensus regarding the benign or malignant diagnosis of the lesions. Cases of metastatic DPN have been reported also by other groups, mentioning that these lesions presented “borderline” or “atypical” features (8–10), hinting at the uncertain malignant potential of these lesions where the diagnosis and therapeutic management is challenging. In the present review, we divided the included studies into two groups, DPN and B-DPN. A total of 355 patients were included in the DPN group and during a follow-up period ranging from 4 months to 23 years only two local recurrences were reported and no metastasis. In the second group, B-DPN, 48 patients were included with 24 patients undergoing SLNB and five block lymph node dissection after positive SLNB, and some patients even receiving adjuvant interferon alpha therapy afterwards. In this “high risk” group the recurrence rate was low, four patients developing local recurrences, and three widespread metastatic diseases. The fact that none of the patients in the DPN group developed metastatic disease and that even though 12 patients from the B-DPN group had positive SLNB only three, two being initially misdiagnosed, developed widespread metastatic disease, highlights the benign behavior of this melanocytic lesion. However, based on the two studies by Magro et al. (8, 9) it should be further evaluated and investigated if a broader initial surgical approach would not prove to be beneficial for patients with borderline/atypical DPN.

Even though the age at diagnosis for DPN and for B-DPN displayed a wide range from 3 months to 64 years, in our review the lesions were usually diagnosed in young individuals before the age of 30 years (1, 6, 8, 9). Congenital DPN have been previously reported in the literature (6) but most DPN are acquired lesions (3). In one case included in our review, the authors report on a DPN developing in a congenital nevus of the right popliteal fossa of a 3-month old boy (14). The initial excision of the lesion showed positive deep margins due to which the patient underwent a re-excision having afterwards no further evidence of recurrence or metastasis during a 15 months follow-up period. The youngest B-DPN case found was the 4-year-old boy initially diagnosed with a DPN but re-diagnosed as a melanoma after a lymph node metastasis was identified 4 months later. The boy did not show any further metastatic disease, however he did undergo a type III modified radical neck dissection with the excision of 39 lymph nodes, all histological negative. The differential diagnosis between a DPN and malignant melanoma in children can be difficult as DPN can demonstrate cytological atypia and presence of mitotic activity, raising suspicion of malignancy (2). The authors mentioned that they considered the diagnosis of a pigmented epithelioid melanocytoma but because not all histologic criteria were met the diagnosis was changed to melanoma (20). It would be interesting to re-evaluate the lesion and determine if applying the criteria mentioned in more recent studies (7–9) the lesion would be better classified as a borderline/atypical DPN.

A recent publication looking at the genetics of melanocytic lesions with histological aspects of DPN has made observations distinguishing this entity from other melanocytic tumors (21). Aside from harboring mutations activating the MAP kinase pathway (i.e., *MAP2K1* or *BRAF*), also observed in other nevi, it became apparent DPN have frequent concurrent *CTNNB1* mutations. This leads to continuous activation of the beta-catenin signaling pathway. This was also observable histologically. In conventional nevi, melanocytes become smaller (“mature”) and show lower expression of CTNNB1 and CCND1 by immunohistochemistry in the deeper dermis. In DPN, the cells do no become smaller (“mature”) and retain expression of CTNNB1 and CCND1 in the lower dermis. The authors also identified that malignant DPN (melanomas arising in DPN) demonstrated additional genetic alterations (to MAP kinase activating and *CTNNB1* mutations). These findings have a number of relevant implications. CTNNB1 immunohistochemistry can be useful to identify DPN lesions (19, 22). Additionally, the mutation profile can aid in differentiating blue nevi (which have *GNAQ* or *GNA11* mutations (23)) from DPN, which can be difficult based solely on histological criteria. Lastly, the presence of additional genetic events such as *TERT* promoter, *CDKN2A, TP53*, or other mutations, argues for a malignant tumor and poor prognosis.

The recently described genetic findings could be of considerable aid in correctly diagnosed DPN and B-DPN. These genetic findings were included in the latest WHO classification system for melanocytic tumors, which placed DPN and B-DPN between common nevi and melanomas in the intermediate/melanocytoma groups, with DPN labeled low-grade and B-DPN a high-grade dysplasia (24). Detailed follow-up studies which apply the genetic criteria presented by Yeh et al. are currently lacking. Of particular value would of course be a larger prospective study. This data could potentially further refine the model proposed by Yeh et al. and allow a genetically based more precise estimation of a tumors behavior in cases where these are difficult to classify based on histopathological assessment alone.

The strength of our review is highlighting a substantial lack of prospective cohort studies combining clinical and histopathological correlation with genetic assessment to identify effective strategies for the clinical management of DPN and B-DPN. At the same time, the low number of included studies is a limitation for an appropriate statistical analysis in the management on DPN and borderline/atypical DPN.

## Author Contributions

IC and TS contributed to the study conception. IC, TS, AT, and LU contributed to the study design. Data collection and analysis was performed by IC and KG. IC, AT, KG, LU, and TS prepared and reviewed the manuscript.

## Conflict of Interest

The authors declare that the research was conducted in the absence of any commercial or financial relationships that could be construed as a potential conflict of interest.
